# Transition to self-management among emerging adults with type 1 diabetes: a mixed methods study

**DOI:** 10.3389/fcdhc.2024.1332159

**Published:** 2024-05-07

**Authors:** Rebecca J. Vitale, Noa Asher, Kaitlyn Shank, Biren Katyal, Liane J. Tinsley, Katharine C. Garvey, Lori M. B. Laffel

**Affiliations:** ^1^ Section on Clinical, Behavioral, and Outcomes Research, Joslin Diabetes Center, Boston, MA, United States; ^2^ Division of Endocrinology, Department of Medicine, Brigham & Women’s Hospital, Boston, MA, United States; ^3^ Division of Endocrinology, Department of Pediatrics, Boston Children’s Hospital, Boston, MA, United States; ^4^ Department of Medicine, Brigham & Women’s Hospital, Boston, MA, United States; ^5^ Department of Pediatrics, Boston Children’s Hospital, Boston, MA, United States

**Keywords:** type 1 diabetes, self-management, young adults, emerging adults, diabetes knowledge, diabetes technology

## Abstract

**Introduction:**

Emerging adulthood is challenging for young people with type 1 diabetes (T1D). This study evaluated transition to diabetes self-management and perceptions of care transfer using mixed methods.

**Methods:**

An online survey queried demographics, management characteristics, diabetes knowledge, self-care readiness, adherence, and diabetes distress. T-tests compared survey scores between those with self-reported target A1c <7.0% versus ≥7.0%. Pearson correlations assessed associations between A1c and diabetes distress, stratified by A1c <7.0% versus ≥7.0%. Qualitative semi-structured interviews elicited perceptions of young adults; transcripts were analyzed using directed qualitative content analysis.

**Results:**

Of 141 participants (30% male, 84% non-Hispanic white) completing the survey, 41% self-reported target A1c <7.0%. Diabetes knowledge and self-care readiness scores did not differ between those with A1c <7.0% versus ≥7.0%, while diabetes distress was lower (45 ± 20 vs 52 ± 20, p=0.01) and adherence higher (77 ± 12 vs 71 ± 14, p=0.02) in those with A1c <7.0% versus ≥7.0%. Diabetes distress was significantly associated with glycemic outcomes in those reporting A1c ≥7.0% (R=0.36, p<0.01). Qualitative analysis (24 participants) revealed five themes and two sub-themes, notable for need for more mental health support, support from others with T1D, benefits of technology for care autonomy, and challenges of obtaining diabetes supplies.

**Discussion:**

Emerging adults with self-reported target A1c endorsed lower diabetes distress and higher adherence than those with elevated A1c. Mental health access, support from others with T1D, technology use, and guidance for supply acquisition may improve transition to self-management and care transfer for emerging adults with T1D.

## 1 Introduction

Emerging adulthood was defined as “a prolonged period of independent role exploration during the late teens and twenties” by Dr. Jeffrey Arnett (1). The early period of emerging adulthood, encompassing ages 18-24, is characterized by “competing educational, social, and economic demands and difficulty accepting responsibility” (2). In people with chronic medical conditions, this period can be particularly challenging as they experience decreased parental support in managing their disease (3). For people with type 1 diabetes mellitus (T1D), in particular, the teenage years are also marked by changes in insulin sensitivity related to the hormonal changes of puberty (4). The combination of these physiological changes with the psychosocial challenges of the late teenage and early emerging adult periods leads to significantly higher blood sugars. In data from 2017-2022, only 17.7% of 13-18-year-olds and 17.4% of 19-25-year-olds were meeting the A1c target of <7.0% (5, 6).

The challenges in meeting glycemic targets among emerging adults with T1D are further complicated by the transfer from pediatric to adult care providers. Given the challenges of identifying and establishing care in a new practice, gaps in care are common in this age group. Individuals with fragmented care have higher blood sugars and are more likely to be hospitalized for their diabetes than those with continuous care (7). Pediatric and adult endocrinologists rarely communicate directly about patients who are transferring, and at times patients arrive to establish care at adult offices with no records and with minimal understanding of their own treatment history, which can make it challenging for the adult provider to provide high-quality care (8).

Many emerging adults with T1D are not satisfied with their experience of the transition to self-management and transfer to adult care. More than one third of patients with T1D reported feeling poorly prepared for transition, and self-reported preparedness was associated with improved glycemic outcomes, while unpreparedness was associated with gaps in care >6 months (9). With the diversity of barriers to care within this patient population, it is important to understand this process from the perspective of emerging adults with T1D themselves.

The aim of this study was to better characterize the experiences of the transition to self-management by associating self-reported survey measures with glycemic outcomes and to deepen our understanding by adding qualitative analysis of emerging adult voices regarding expectations of self-care and transfer to adult providers. The use of validated survey measures to help quantify key constructs provided a sound methodology to address this aim. The addition of qualitative methodology offered a unique opportunity to understand emerging adults’ perceptions, beliefs, and expectations regarding transition.

## 2 Materials and methods

To address the study aim, a mixed methods approach was used with both qualitative and quantitative components, to allow for a multidimensional exploration of this critical phase of T1D management.

### 2.1 Quantitative

Participants were recruited from one pediatric endocrinology center and one center with both pediatric and adult endocrinology care. The quantitative portion of the study was approved as exempt by the Joslin Diabetes Center’s and Boston Children’s Hospital’s Institutional Review Boards prior to participant recruitment. A list of patients with T1D ages 18-25 years who had been seen in the clinics within the last year was generated from the electronic medical record. Secure recruitment emails that included a link to the online survey were sent to all individuals with a valid email address who met these criteria. The link directed participants to a page describing the study on the RedCap electronic data capture tools hosted at Joslin Diabetes Center (10, 11). REDCap (Research Electronic Data Capture) is a secure, web-based software platform designed to support data capture for research studies, providing 1) an intuitive interface for validated data capture; 2) audit trails for tracking data manipulation and export procedures; 3) automated export procedures for seamless data downloads to common statistical packages; and 4) procedures for data integration and interoperability with external sources. Inclusion criteria were listed as: age 18-25 years, having had a diagnosis of type 1 diabetes for more than 1 year, and ability to speak and read English. Participants who met these criteria via self-report were able to advance to complete the survey. Participants who completed the survey were directed to a separate survey to enter their information to receive a $10 e-gift card to thank them for their participation.

The online survey included a demographic and diabetes management questionnaire, a validated measure of self-care readiness (the Readiness for Independent Self-Care Questionnaire, RISQ) (12), a validated measure of adherence to the care plan (the Diabetes Management Questionnaire, DMQ) (13), and a validated measure of diabetes distress (the Problem Areas in Diabetes – Emerging Adult Version, PAID-EA) (14), and a measure of diabetes knowledge that was developed for this study. All diabetes management and outcomes data were collected by self-report. The RISQ is a 20-item self-report questionnaire of diabetes self-management. Higher scores (scale 0-100) indicate greater readiness for self-management. The DMQ is a 21-item self-report questionnaire of adherence to the care plan. Higher scores (scale 0-100) indicate greater adherence. The PAID-EA is a 25-item questionnaire of diabetes distress. Higher scores (scale 0-100) indicate greater distress.

The diabetes knowledge measure was a 43-item questionnaire that included questions on diabetes treatment goals, hyperglycemia and sick day management, diabetes complications, hypoglycemia, exercise, and nutrition. The questions were developed by physicians familiar with diabetes management in young adults (R.J.V., K.C.G., and L.M.L.) and were based on questions from other validated or clinically-used measures (15, 16). The measure was reviewed by multiple physicians and certified diabetes care and education specialists prior to being administered. The score represents the percent of questions answered correctly, so higher scores (scale 0-100) indicated greater diabetes knowledge.

Statistical analyses were performed using SAS software, Version 9.4 of the SAS System for Windows (SAS Institute, Inc., Cary, NC, USA). Surveys in which all measures were ≥75% complete were included for analysis; duplicate survey entries (23 entries) were excluded. Characteristics of participants who reported meeting the A1c target of <7.0% were compared with those not meeting the target (A1c ≥7.0%) using t-test, chi-square test, and Fisher’s exact test as appropriate. Diabetes knowledge, RISQ, DMQ, and PAID-EA scores were compared among those who were and were not meeting the glycemic target using t-tests. Multivariable generalized linear models were conducted, including the baseline characteristics found to be different between those meeting and not meeting the glycemic target, to evaluate whether their inclusion impacted the significance of the results.

On evaluating plots of survey scores with A1c, correlation between A1c and diabetes distress appeared to be bimodal, leading to stratified analysis. Pearson correlation coefficients between A1c and PAID-EA score were calculated separately for those with A1c <7.0% and those with A1c ≥ 7.0%.

### 2.2 Qualitative

Participants were recruited from two pediatric endocrinology clinics. The qualitative portion of the study was approved by the Joslin Diabetes Center’s and Boston Children’s Hospitals’ Institutional Review Boards prior to participant recruitment. Inclusion criteria included ages 18-25 years, having had a diagnosis of type 1 diabetes for more than 1 year, and ability to speak and read English. Exclusion criteria included inability to provide informed consent, not having had a diabetes clinic visit within the last year, and significant developmental or cognitive disorder. A list of patients with T1D age 18-25 years who had been seen in the clinics with in the last year was generated from the electronic medical record. The study was advertised to providers in both clinics, and they identified patients under their care who would be amenable to the study. The study team approached these individuals via phone or secure email. Towards the end of the study, purposive sampling was used to recruit individuals with higher A1c, diverse those with racial/ethnic backgrounds, and non-insulin pump users to ensure that these perspectives were represented in the data.

A structured interview guide was created with input from a team including pediatric and adult endocrinologists and nurse practitioners as well as behavioral health and transition experts. In addition, the proposed questions underwent cognitive debriefing with members of the target audience to ensure their appropriateness. The interview guide applied open-ended questions to elicit participant experiences with diabetes, knowledge about diabetes, and experiences with and concerns regarding transition to self-management and transfer of care. The interviewer (N.A.) was highly familiar with the intended participant group and received training in qualitative interview techniques by the research group. The interviews were conducted virtually via HIPAA-compliant Microsoft Teams videoconferencing software. All participants provided informed consent prior to the interview. Participants received a $40 e-gift card to thank them for their participation.

Participants were asked closed-ended demographic and diabetes management questions at the beginning of the session prior to the start of the semi-structured interview. Additional demographic and diabetes management data was gathered from the electronic medical record after the interview session.

Interviews were transcribed using a secure third-party service. De-identified interview transcripts were analyzed using directed qualitative content analysis (17). Interviews were coded by authors with significant personal and professional experience with T1D (R.J.V., K.S., and B.K.). Two-to-three coders read transcripts of each interview and coded independently. The authors used iterative analytic memoing to guide the analysis. The codes were compared, with the third coder available to adjudicate disagreements when only two coders had reviewed the transcript. The codes were placed into categories and subcategories, which were organized into themes.

## 3 Results

### 3.1 Quantitative

The survey was completed by 141 participants with mean age 21.5 ± 2.2 years (**Table 1**). 30% of participants identified as male, 84% identified as non-Hispanic white, and 57% were students. The majority (78%) were covered by their parents’ insurance, while 13% had their own private insurance plan and 8% were using public insurance. Diabetes duration was 12.2 ± 5.4 years, and the majority of participants (89%) were still receiving care from a pediatric provider. The mean self-reported A1c was close to target at 7.3 ± 0.9%, with 41% reporting a target A1c of <7.0%. Diabetes technology use was high, with 79% using insulin pumps (68% hybrid-closed loop) and 93% using continuous glucose monitors (CGM). Mean self-reported time in target range (TIR) 70-180 mg/dL among CGM users was 61 ± 17%.

**Table 1:** Survey participant characteristics by glycemic outcomes.

	Total Sample (N=141)	A1c <7.0 (n=55)*	A1c ≥ 7.0 (n=80)*	p-value**
Age (years)	21.5 ± 2.2	21.8 ± 2.3	21.3 ± 2.1	0.21
Gender (% Male)	30%	29%	31%	0.80
Race (% NHW)	84%	82%	85%	0.62
Student status (%)	57%	55%	58%	0.67
Insurance status				0.88
% parents insurance	78%	78%	79%	
% private insurance	13%	11%	14%	
% public insurance	7%	9%	6%	
Diabetes duration (years)	12.2 ± 5.4	**10.5 ± 6.0**	**12.9 ± 4.6**	**0.01**
Pediatric care (%)	89%	85%	91%	0.30
A1c (Self-report, %)	7.3 ± 0.9	**6.5 ± 0.3**	**7.8 ± 0.8**	**<0.01**
TIR (among CGM users, %)	61 ± 17	**69 ± 13**	**54 ± 17**	**<0.01**
Insulin pump use (%)	79%	**89%**	**73%**	**0.03**
Hybrid-closed loop pump use (%)	68%	75%	63%	0.08
CGM use (%)	93%	96%	93%	0.47

*6 participants did not report A1c.

**p-values from t-test (age, diabetes duration, A1c, TIR), chi-square test (gender, race, student status, pediatric care, insulin pump use, hybrid-closed loop pump use), or Fisher’s exact test (CGM use, insurance status).

CGM, continuous glucose monitor; TIR, time-in-range on continuous glucose monitor.

Values are bolded if p<0.05.

When comparing those participants with A1c <7.0% (n=55, 39%) to those with A1cs ≥7.0%, those meeting the target had a shorter duration of diabetes (10.5 ± 6.0 years vs 12.9 ± 4.6 years, p=0.01), were more likely to use insulin pumps (89% vs 73%, p=0.03), and had higher TIR among those using CGM (69 ± 13 vs 54 ± 17, p<0.01). There were no differences in age, gender, race, student status, insurance status, clinic setting (pediatric vs adult), hybrid-closed loop pump use, or CGM use (**Table 1**).

The participants completed four measures: diabetes knowledge, adherence (DMQ), self-care readiness (RISQ) and diabetes distress (PAID-EA). The results of all measures (including the newly-developed diabetes knowledge measure) are scored from 0-100 with higher scores indicating greater endorsement of the survey construct. Participants scored highly on the diabetes knowledge measure, with mean score of 89 ± 8. Interestingly, there was no significant difference between those meeting and not meeting glycemic target (**Table 2**). DMQ scores did differ by glycemic target; the mean score was 73 ± 13; those meeting the target had a score of 77 ± 12 while those not meeting the target had a score of 71 ± 14 (p=0.02). RISQ scores were high among all participants (mean 90 ± 9) and did not differ between those meeting and not meeting the glycemic target. Diabetes distress (mean PAID-EA score 50 ± 21) was higher among those not meeting the glycemic target (54 ± 20) than those meeting the target (45 ± 20, p=0.01). Insulin pump use and diabetes duration, which were found to be different between those meeting and not meeting the glycemic target, were not significantly associated with PAID-EA or DMQ scores when included in generalized linear models that also included a categorical measure of A1c <7.0% vs ≥7.0%. Additionally, the addition of these variables did not affect the significance of the relationship between adherence and A1c or diabetes distress and A1c.

**Table 2:** Survey results by glycemic outcomes.

	Total Sample (N=141)	A1c <7.0 (n=55)*	A1c ≥ 7.0 (n=80)*	p-value**
Diabetes Knowledge	89 ± 8	90 ± 6	88 ± 9	0.12
Adherence (DMQ)	73 ± 13	**77 ± 12**	**71 ± 14**	**0.02**
Self-Care Readiness (RISQ)	90 ± 9	91 ± 6	89 ± 10	0.26
Diabetes Distress (PAID-EA)	50 ± 21	**45 ± 21**	**54 ± 20**	**0.01**

*6 participants did not report A1c.

**p-values from t-test.

Values are bolded if p<0.05.

**Figure 1:**
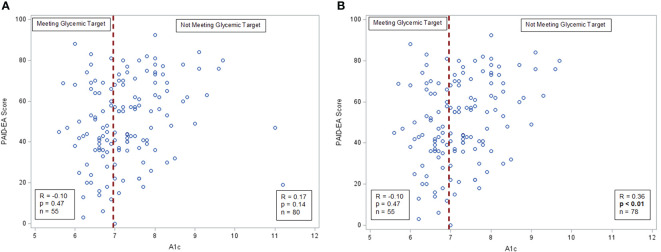
A1c and diabetes distress correlations. **(A)** A1c and distress among all participants, Pearson’s correlation. **(B)** A1c and distress with outliers removed, Pearson’s correlation.

On reviewing the correlations between A1c and the scores of the measures listed above, it was noted that diabetes distress appeared to have a bimodal distribution, with those with A1c <7.0% having a broad range of PAID-EA scores while those with A1c ≥ 7.0% appeared to have a positive correlation between A1c and distress (**Figure 1**). Pearson’s R for those with A1c <7.0% was -0.10 (p=0.47) and for those with A1c ≥7.0% was 0.17 (p=0.14). There were two outliers with A1c ≥11.0 who had lower-than-expected distress. Given that A1c and TIR were strongly correlated (Pearson’s R -0.51, p<0.01) we sought to validate these A1c values by comparing to TIR, however neither participant reported a TIR value. When these outliers were removed, Pearson’s correlation for those with A1c ≥7.0% was significant with r=0.36 (p<0.01).

### 3.2 Qualitative

The qualitative interview involved 24 participants with a mean age of 21.5 ± 1.8 years (**Table 3**). 46% were male, 42% female, and 13% identified as trans or non-binary, and 75% identified as non-Hispanic white. The mean age at diagnosis was 10.0 ± 6.3 years. All participants received their diabetes care in the pediatric setting. A1cs were lower than average in this sample, with 71% having an A1c <8%, and there were high rates of diabetes technology use with 83% using insulin pumps and 96% using continuous glucose monitors. Diabetes complications were rare, but 13% of participants did have evidence of microalbuminuria.

**Table 3:** Interview participant characteristics.

	Total Sample (N=24)
Age (years)	21.5 ± 1.8
Gender (%)	
Male	46%
Female	42%
Trans or Non-Binary	13%
Race (% NHW)	75%
A1c < 8% (%)	71%
Age at diagnosis (years)	10.0 ± 6.3
Insulin pump use (%)	83%
Continuous Glucose Monitor use (%)	96%
Microalbuminuria (%)	13%

The interview guide focused on the concrete steps that were necessary to successfully self-manage diabetes, allowing participants to identify specific facilitators and barriers to self-management. Participants reflected on their experiences in the transition to self-management of T1D, identifying three important facilitators of transition, one barrier to transition, and one feature of pediatric care that was particularly valued. In total, five themes and two sub-themes emerged from the data.

#### 3.2.1 Theme 1: young adults value the support of others with T1D

Many participants reflected on their experience that T1D impacts all aspects of their lives, and has become integrated into their identity. They found that relationships with others who have T1D themselves, both inside and outside the healthcare team, can be a crucial support in the transition to self-management. Even while appreciating these individuals, several participants expressed a wish to have more of this type of support:

I actually have … one pretty close friend from high school who has diabetes, and sometimes I’ll talk to her about it … it makes me feel better … [to have] people I know well and care about that I can talk with about it. And they understand and also like to see if … they struggle with … certain aspects of diabetes care. But I largely just feel … very isolated with it, specifically just on the experience of living with diabetes because I just don’t think people understand diabetes well.​ (Male, age 22)

One participant identified relationships with others with T1D as the resource that would help her the most as she started to manage her diabetes on her own:

Maybe more … community with like other similar aged patients with diabetes would be nice…. Sometimes I wish I had more … friends who had diabetes … that would be kind of a good way to also like help people with growth or … independence with everything is if they had more peers … connecting a little more. (Female, age 20)

These relationships do not need to be in-person. Multiple participants discussed the importance of social media, particularly the online community of Reddit, as a go-to source of diabetes-related information and support:

One of my favorite resources is … a type 1 diabetic [sic] Reddit thread…. There’s like a little corner of the chat for you with any problem that you might have. Somebody’s talked about it before, whether it’s…“My skin gets irritated by adhesive from my pump,” or… “I think I’m allergic to my cannula because of the way my skin flares up,” just crazy stuff that you’d like never even consider, there’s people are in there talking about it. …when I discovered that, that was like a whole new world. It’s a huge resource. (Male, age 22)

Emerging adults with T1D trust others who have had similar experiences, and others with T1D become important sources of information and support as they develop independence in diabetes management.

#### 3.2.2 Theme 2: technology can promote autonomy

When discussing facilitators of self-management, the idea that technology, both general and diabetes-specific, can promote independence emerged repeatedly. Technology can simplify diabetes management in a variety of ways, which reduces the burden on the individual as they consolidate those tasks and rely less on their caregivers for help. For some people, it was diabetes technology itself that was most useful:

…a new sensor and a new pump meant all new equipment from what I was used to. I was like, ‘Alright, this is my chance. I’m starting something completely new. The best way to go about doing it is to do it myself,’ and that’s kind of when I took things over. (Male, age 24)

Several other participants identified other forms of technology as being particularly helpful in diabetes management, such as phone apps for communication with their providers or pharmacies:

My dad would [refill prescriptions] for me all the time, but I actually started doing that … it’s because I got the CVS app, and then I started … ordering stuff all on my own instead of waiting for them to call me. And then I was like, “This is a lot better for me”…being able to easily order my prescriptions when I need them instead of having to … call. I used to have to … e-mail or message [my providers], “I need this prescription refilled or whatever,”… [but] instead, I can request it on the CVS app. (Male, age 22)

By making specific aspects of diabetes management easier, such as glucose monitoring, insulin delivery, or refilling prescriptions, these technological advances decrease the overall load of diabetes management and make it more possible for an individual to manage independently.

#### 3.2.3 Theme 3: young adults desire more mental health support during the transition period

Multiple participants discussed the importance of mental health support, which they felt was under-addressed in their experience of T1D care:

…diabetes is a condition that can cause a lot of like mental health stress, and just overall it’s very hard to manage sometimes, and it can impact your mental health a lot. I think a lot of mental health issues might not be getting caught or might be getting a little bit swept under the rug just because [providers are] not as focused on that in … appointments. (Female, age 20)

One participant reinforced the relationship between mental health challenges and burnout-related disengagement with care, noting that when he had challenges in his mental health, he was less likely to take care of his diabetes.

I think that diabetes [care] could benefit from more focus on discussions around mental health with patients … when I’m feeling like worse about myself, I immediately stop … caring about … checking my blood sugar, or it’s easier for me to forget to take insulin…. It’s not just diabetes. It’s just like taking care of anything in my life at that point feels hard … I’ve struggled with … depression and anxiety my whole life. Like that’s not really new to me. …I just think it would help [to have] the opportunity … of having like an outlet for it…. (Male, age 22)

While burnout can be challenging at any time of life, the transition period is characterized by changing social supports and decreased supervision from caregivers, leading to an even greater impact on diabetes-related health.

#### 3.2.4 Theme 4: individualized care in pediatric setting is valued

In addition to describing their experiences with the transition to self-management, participants also discussed their concerns about the transfer to adult care. Most participants reported some degree of anxiety about the idea of seeing an adult diabetes provider, and this led them to reflect on the aspects of care that they valued from their pediatric providers. Some participants preferred making long-term plans and goals with their providers, “[My endocrinologist] plans it out ahead … six months, a year, so I feel a lot more confident leaving with a long-term goal [to work] towards,” (Male, age 20). Others wanted to dive into their glucose trends and look through the day-to-day minutiae: “…it is helpful … going into more like nitpicking through the numbers and discussing the numbers and making changes,” (Female, age 25).

Some other participants appreciated the way their team provided tailored communication:

“I have a team that knows me and that I can communicate very well with, especially because I have a very odd way of communicating as a … neurodivergent person … it means that I can make the changes needed and those changes are able to be like explained in a way that make sense to me.” (Trans male, age 23)

Nearly all of the participants in the study identified a specific aspect of their pediatric provider’s approach to care that was valuable to them. In some cases these represented opposite approaches to care reported by participants with the same provider. This suggests that these providers were able to ascertain the approach that would be most successful for the patient, and by doing so provided an individualized approach to care. Participants were concerned that adult providers would take a more “one-size-fits-all” approach to diabetes care, with less personalization. This core concern seemed to underlie many of the participants’ worries about having to build new relationships with adult providers, which they associated with their hesitance to transfer to adult care.

#### 3.2.5 Theme 5: self-management skills are acquired in a consistent order

There was a striking consistency in the order in which participants reported gaining self-management skills. Independence in direct self-care, such as monitoring blood glucose and administering insulin, always came first, followed by indirect management tasks as a separate category, “I feel like there’s two arms of the independence. There’s … the administrative independence and … the care independence,” (Woman, age 23).

Among the indirect management tasks, communicating with providers and booking appointments were the first steps, with ordering and obtaining diabetes supplies from pharmacies and durable medical equipment (DME) providers as the second. The final step was managing communication with the insurance company and working through insurance challenges; even among these 18-25 year old participants, very few had taken over this aspect of diabetes care:

I feel like the only remaining thing for me to be … completely independent is … insurance. When I was in like middle school or early high school … I like definitely transitioned to … handling the day-to-day stuff myself … my mom was not helping me do any of that anymore and … now I … pick up most of my supplies from like the pharmacy and stuff, sometimes my mom does.…I go to all the appointments myself … I call my doctors myself, I make my appointments … so I think being completely independent would be like all that stuff plus handling like insurance and I don’t know like contacting … durable medical equipment [suppliers] if there are issues … my mom still does that so once I do that then I will be like fully independent. (Woman, age 20)

One participant noted that even though he was “mostly independent” with both direct and indirect management tasks, he still shares tasks with others in his life who are supporting him in diabetes management:

I think I’m mostly independent right now….for a long time I think I relied on my parents’ help to … schedule appointments, do insurance stuff, and … advise on care and stuff like this, but I think I also always wanted to be more independent. And now that I’m on my own insurance … I think now I’m pretty much independent … you reach out to the right people when you have questions or need help. You … take care of your own … supplies, scheduling, all that, and … share with the people that care about you … my significant other, my family, what’s going on so that they can also help you manage your diabetes. (Male, age 24)

His definition of independence includes reliance on others, specifically knowing who to reach out to for help. The group of people supporting his diabetes has expanded to include his significant other, and he acknowledges that even in independence it is important that the people who care about him are knowledgeable about his diabetes as well, so that he has the help he needs to manage his T1D.

#### 3.2.6 Subtheme 1: independence requires awareness of complexity

While even “independent” adults with diabetes require a support system, achieving appropriate independence in diabetes management relies on the awareness of its many complex components, which may vary between individuals. Most of the participants expressed awareness of the scope and complexity of the indirect management tasks necessary for optimal diabetes management, but several younger participants felt that these tasks would not be difficult to master:

…whenever I get my own … medical insurance and I can get my stuff, that is what I view as independent. I mean as far as waking up on my own when I am low, waking up when my blood sugar is high, I wake up to my alarms I don’t really have a problem with … handling it independently….[my mom] is the insurance holder so there is nothing I can really do about that, but once I get [my own] insurance I’m sure I will be okay … I mean it is just making a call. I don’t really feel like that is going to be too much of a problem. (Male, age 19)

The nonchalant way in which he describes dealing with insurance companies suggests that he has never directly engaged with this process, especially when compared with descriptions of this process from participants who had more direct experience:

…[it’s] horrible every time. I don’t know, there’s long hold times, and it feels like every time you call them, they’ll tell you something different. They can never seem to explain it to me in a way that makes sense, and I don’t feel like I’m a stupid person, so I don’t understand why it doesn’t make sense to me. …my pump went out of warranty recently, so I had to go through the process of getting a new one, and like I think the whole pump is going towards my deductible, but then there’s also like a copay – no, no, no, it’s not a copay because a copay would be a fixed amount. It’s a coinsurance, so it’s like some ridiculous sum, but nobody can explain to me why my like infusion sites do not go towards my deductible, but suddenly when I’m getting a pump, it does. Like that makes no sense to me….you end up spending like two hours on the phone, and then I ended up on some weird three-way conference call between like [insurance company] and [DME supplier] and me, and like the guy was providing the weirdest reasons that I simply could not understand….I just feel like it’s very frustrating every time a call, and I never get a straight answer … every time I called them, I feel like they were telling me different places. They were telling me different processes, and it just feels like this cycle where you cannot get a straight answer…. (Female, age 23)

All participants who had personally engaged with their insurance companies regarding coverage issues described the experience as being very negative. Those younger participants who had not yet made such calls on their own were sometimes unaware of the difficulties they could expect to encounter.

#### 3.2.7 Subtheme 2: obtaining supplies is the most challenging part of management

The challenges of day-to-day management of T1D are often overshadowed by the numerous barriers to supply and medication acquisition, especially as compounded by insurance coverage. The most common responses to the interview guide prompt “what is the hardest thing about managing your diabetes” related to acquisition of medications and supplies from pharmacies and DME suppliers:

“Honestly reordering and … juggling supplies … getting my Dexcom sensors … trying to order my pump supplies and … insulin … I have to get them from different places and somehow it never works out with my insurance. I’m always like missing something which is kind of frustrating” (Female, age 21).

One participant described feeling completely overloaded by the conflicting priorities of managing diabetes while also keeping up with the rest of their life as a college student: “I’m busy 24/7, and so then I have no executive function left for things like managing medical supplies. …what I think they should actually do is like be like, ‘Hey, your supplies are running low … you should refill it’” (Non-binary, age 22).

This participant is looking for help organizing themselves and finding the time to successfully acquire supplies. Some participants described their provider teams as being very supportive in this area:

I guess if anything, like the resources are important … the help with the more difficult … intricacies … like insurance and … sourcing your supplies and stuff like that. The team at [medical practice] is … fantastic about helping with that, and that’s really helped me out along the way … I have three forms of insurance, and I don’t know anything about any of them … [my provider] has helped me a million times with … prior authorizations, sorting out when my insurance … won’t fill something….that has been a huge help because that is like a ton of time … just sitting there. You’re on hold for like an hour to talk to these people, and … at this point in life, I just don’t have that kind of time. (Male, age 22)

This participant, like the participant above, describes having difficulty managing competing priorities between diabetes-related tasks and other life tasks. Emerging adults with T1D have identified this as a significant difficulty in their lives, and they often look to their providers for help with these tasks. While this is somewhat outside the typical model of medical care in diabetes, this is a crucial way for providers to support their emerging adult patients, especially those who are navigating these issues for the first time.

## 4 Discussion

This mixed methods study aimed to better understand perceptions of young adults as they acquire self-care and prepare for transfer from pediatric to adult diabetes care settings. In combination, we sought to evaluate associations of diabetes knowledge, adherence, self-care readiness, and diabetes distress with glycemic outcomes assessed as self-reported A1c, as well as perceptions of challenges and opportunities to enhance transition to self-management and care transfer using qualitative methods.

As expected, greater reported adherence was related to lower A1c. Further, diabetes distress was strongly and directly associated with glycemic outcomes. However, it was notable that that diabetes knowledge did not differ between those meeting and not meeting the A1c target of 7.0%. Additionally, perception of self-care readiness did not differ in those at target versus above A1c target. The relationship between distress and A1c seemed to differ between those meeting and those not meeting the glycemic target. Of note, some young adults reporting optimal glycemic outcomes also reported elevated levels of diabetes distress. The importance of diabetes distress and the mental burden of T1D was reinforced in the qualitative data, in which the desire for increased mental health support was a prominent theme. Participants were able to identify a variety of factors that were helpful in supporting the transition to self-management, including support of others with T1D, technology use, and support with the complex process of supply acquisition. Providing the transition support that emerging adults are requesting could in fact help to improve diabetes distress, as the qualitative data shows that some of these issues are significant sources of distress.

The lack of association between diabetes knowledge and A1c target achievement is likely multi-factorial. Many transition interventions over the last few decades have focused on diabetes education as a key component to facilitating self-management and transfer of care among emerging adults, and indeed many of them have been successful in transiently lowering A1cs (18–21). Sequeira et al. devised a structured transition program with targeted as well as group diabetes education that led to lower A1c after 12 months when compared with those receiving usual care (21). Diabetes knowledge seems to be necessary for successful self-management of T1D, but these data suggest that knowledge alone is insufficient to optimize glycemic outcomes. Knowledge does not always translate to behavior. The goal-directed behaviors needed to complete diabetes-associated tasks rely on skills such as planning and organization, which are components of executive function (22). Notably, executive function skills are still developing in emerging adulthood, which may partly explain the gap between knowledge and behavior in this developmental stage.

Diabetes distress, as measured in the quantitative portion of the study by the PAID-EA, was significantly associated with A1c, with higher diabetes distress seen in those with A1c ≥7.0%. Our qualitative results also supported the importance of diabetes distress; while the phrase “diabetes distress” was not specifically used, many participants discussed the mental burden of diabetes and a desire for mental health support was one of the five themes. One 22-year-old male participant described the relationship between mental health and diabetes as “when I’m feeling like worse about myself, I immediately stop … caring about … checking my blood sugar, or it’s easier for me to forget to take insulin,” which is very similar to definitions of diabetes distress and burnout (23). Both arms of this mixed methods study highlight the importance of addressing mental health and diabetes distress throughout the transition to self-management and transfer to adult care. The THR1VE study evaluated a positive psychology SMS text messaging intervention to treat diabetes distress in teens age 13-17 and found that participants had high engagement with the intervention (24), but the impact of this intervention on distress has not yet been analyzed. Future interventions for emerging adults with T1D should address distress as well as diabetes education.

Further evaluation of the relationship between diabetes distress and A1c revealed a bimodal distribution. People meeting the glycemic target had a wide range of scores on the PAID-EA, from 3 to 88 (on a 0-100 scale, with higher scores reflecting more diabetes distress), and there was no significant correlation between A1c and PAID-EA score in this group. When the outliers were removed, those with A1c ≥ 7.0% did demonstrate a significant association between distress and A1c, with participants with higher A1cs reporting higher levels of diabetes distress. High levels of diabetes distress, however, are not limited to people with high A1cs; many participants who were meeting A1c targets had high levels of distress. While A1c and CGM TIR are measured as primary outcomes in the majority of diabetes studies, it is important to remember that those meeting glycemic targets may still benefit from distress interventions. Indeed, the diabetes distress in the population of individuals meeting glycemic targets could possibly differ from that experienced by people not meeting glycemic targets, and this is an important area for future inquiry.

Multiple participants in the qualitative arm of the study reported that supply acquisition and other indirect management tasks are the most difficult parts of diabetes management, and anxiety around these issues likely contributes to diabetes distress. Challenges with insurance coverage for diabetes supplies are increasingly common, and the high financial burden for people with diabetes has been described, with ongoing investigations into the impact on people with diabetes (25–27). Participants in this study, however, focused on complexity of diabetes supply acquisition rather than the financial burden. The administrative burden of the increasingly-complicated insurance structure in the United States has been described as it relates to providers (28), but the impact of these challenges on patients has received less attention in the literature. Participants requested more support in this area from their providers, who may have limited understanding of the intricacies of a particular insurance plan themselves. Blanchette et al. have developed a Financial Toolkit to help provide assistance in navigating financial and insurance issues for people with T1D, which could be a valuable resource in this endeavor (29). This toolkit is focused on the financial aspects of supply acquisition, however, and based on our qualitative findings, emerging adults with T1D may additionally benefit from assistance with organization and planning around supply management. The development of such tools is an important area for future research.

Independence from parental support in diabetes care is an important goal to pursue for emerging adults, but it is crucial to acknowledge that emerging adults will still need support in diabetes care. Several participants in the qualitative study described independence as knowing who to ask for help, and underscored the importance of “shar[ing] with the people [who] care about you … so that they can also help you manage your diabetes.” The need for support in diabetes management is not limited to children and adolescents. Providers may be able to assist in the transition to self-management by helping emerging adults identify new support people for their diabetes as their parents’ involvement appropriately decreases. It is important to identify support people who are likely to be beneficial, as the involvement of loved ones in diabetes care is not always positive. In evaluating family/friend involvement among adults with type 2 diabetes, one framework differentiates between helpful and harmful involvement (30). Harmful involvement is significantly associated with increased diabetes distress, so these new support people need to be chosen carefully (31).

The use of diabetes technology was quite high among participants in both arms of the study, so it is difficult to draw conclusions comparing those who do and do not use technology. Still, many participants in the qualitative study that were using technology felt that the technology itself had helped to promote independence in self-management. Interestingly, this definition expanded beyond diabetes-focused technology, and included phone apps for pharmacy refills and patient portals that facilitated communication with the care team. Another qualitative study of emerging adults with T1D also highlighted the challenges of communication with pharmacies and care teams (27). Our study confirms those findings and expands upon them by showing that technological means of communication can help to improve this process and support independence among emerging adults. The availability of patient portals and pharmacy apps may be important features for some emerging adults to consider when choosing a pharmacy and an adult endocrinology practice.

Many of the qualitative participants receiving pediatric care were anxious about the impending transfer to adult care, as they felt that the care model would be different in an adult care setting. Nearly all participants remarked on some feature of their pediatric care team that they really valued, and these specific features were often quite different. While one participant appreciated that his provider focused on the “big picture” and future planning, another participant valued the in-depth dives into CGM tracings. These participants both received care from the same provider, which underscores the individualized care approach utilized by this provider. Through their years of experience with each patient, the provider has assessed which clinical approaches are most effective, and then utilizes the appropriate approach. All of the qualitative participants were receiving care in the pediatric setting, so their concerns about more generic care in the adult care setting were hypothetical rather than experience-based. Adult providers may benefit from scheduling longer or more frequent visits with their patients who are being transferred from pediatric care, or from having direct conversations with pediatric providers, in order to provide individualized care. Adult endocrinologists have endorsed that seeing young adults with type 1 diabetes requires more time and resources than older adult patients (8), and perhaps both the patients and the providers would benefit from some of that additional time being spent to get to know one another directly. As the personal relationship appears to be an important aspect of emerging adults’ engagement with their diabetes care team, this is likely a worthy investment of time.

This study did have limitations. The quantitative study’s sample may not be reflective of the general population of emerging adults with T1D given the lower-than-average A1c and high use of diabetes technology among this group, which may limit the generalizability of the study’s findings. Additionally, the majority of participants were non-Hispanic white and female. All people who receive care in these clinics were offered the same opportunity to participate, but those individuals with lower A1cs were more likely to participate and engage with this research. While somewhat more diversity was achieved in the qualitative study by utilizing purposive sampling, this group also had lower A1c and higher technology use than the general population of people with T1D. All of the quantitative data, including glycemic outcomes, were collected by self-report, which introduces the possibility of bias. Wu et al. evaluated the accuracy of self-reported A1c and found that the positive-predictive value of accurately reporting an A1c in the correct range was between 67.5% and 87.7%, and that factors such as gender, age, T1D duration, technology use, socioeconomic status, and depression level did not differentially impact A1c accuracy (32). Given this, it seems likely that the self-report A1c measure, which was most important to the analyses, was relatively accurate. It must also be acknowledged that the measure of diabetes knowledge was not a validated measure, but the current measure was intended to reflect current management practices in the current environment of advanced diabetes technologies.

This study’s strengths include the mixed methods approach, with qualitative interviews allowing for more in-depth and nuanced exploration of the findings in the quantitative portion of the study. We recruited from multiple clinics, though both were within academic centers, so the findings are not reflective of the practice within a single center. The email-based recruitment of the quantitative study allowed for all people with T1D receiving care in these clinics to be offered an opportunity to participate.

In this mixed methods study, we have confirmed the relationship between diabetes distress and glycemic outcomes in emerging adults with T1D, further exploring this relationship to highlight the wide range of diabetes distress seen among people with optimal glycemic outcomes. In qualitative analysis, our emerging adult participants have identified a variety of factors important in transition to self-management, including technology, support of others with T1D, mental health support, and assistance with obtaining supplies. In future studies, we hope to develop and evaluate tools to assist with diabetes supply management as well as better understand diabetes distress among people with T1D who have optimal glycemic outcomes. It would be interesting to conduct a similar qualitative study among emerging adults who have completed the transfer to adult care, to compare the perspectives of adult care endorsed here to perspectives from those who have actually experienced the transfer. We also hope to develop and test interventions to improve the process of the transition to self-management that include those factors our participants have identified to be most important in their experience of transition.

## Data availability statement

The raw data supporting the conclusions of this article will be made available by the authors, without undue reservation.

## Ethics statement

The studies involving humans were approved by Joslin Diabetes Center Committee on Human Studies and the Boston Children’s Hospital Institutional Review Board. The studies were conducted in accordance with the local legislation and institutional requirements. The quantitative portion of the study was found to qualify as exempt and did not require written informed consent. The participants in the quantitative portion of the study provided their written informed consent to participate in this study.

## Author contributions

RJV: Conceptualization, Formal analysis, Funding acquisition, Investigation, Methodology, Project administration, Supervision, Writing – original draft, Writing – review & editing. NA: Investigation, Writing – review & editing. KS: Formal analysis, Writing – review & editing. BK: Formal analysis, Writing – review & editing. LT: Data curation, Formal analysis, Writing – review & editing. KG: Conceptualization, Methodology, Resources, Supervision, Writing – review & editing. LL: Conceptualization, Methodology, Resources, Supervision, Writing – review & editing.
